# Resting State Functional Connectivity of Dorsal Raphe Nucleus and Ventral Tegmental Area in Medication-Free Young Adults With Major Depression

**DOI:** 10.3389/fpsyt.2018.00765

**Published:** 2019-01-25

**Authors:** Amit Anand, Stephen E. Jones, Mark Lowe, Harish Karne, Parashar Koirala

**Affiliations:** ^1^Center for Behavioral Health, Cleveland Clinic, Cleveland, OH, United States; ^2^Radiology Institute, Cleveland Clinic, Cleveland, OH, United States

**Keywords:** ventral tegmental area (VTA), dorsal raphe, young adults, major depression (MDD), resting state–fMRI, functional connectivity, brain connectivity, resting state

## Abstract

**Background:** This study has, for the first time, investigated the dorsal raphe nucleus (DRN) and ventral tegmental area (VTA) resting state whole-brain functional connectivity in medication-free young adults with major depression (MDD), at baseline and in relationship to treatment response.

**Method:** A total of 119 subjects: 78 MDD (24 ± 4 years.) and 41 Healthy Controls (HC) (24 ± 3 years) were included in the analysis. DRN and VTA ROIs anatomical templates were used to extract resting state fluctuations and used to derive whole-brain functional connectivity. Differences between MDD and HCs were examined, as well as the correlation of baseline Hamilton Depression and Anxiety scale scores to the baseline DRN and VTA connectivity. The relationship to treatment response was examined by investigating the correlation of the percentage decrease in depression and anxiety scale scores with baseline connectivity measures.

**Results:** There was a significant decrease (*p* = 0.05; cluster-wise corrected) in DRN connectivity with the prefrontal and mid-cingulate cortex in the MDD group, compared with the HC group. DRN connectivity with temporal areas, including the hippocampus and amygdala, positively correlated with baseline depression scores (*p* = 0.05; cluster-wise corrected). VTA connectivity with the cuneus-occipital areas correlated with a change in depression scores (*p* = 0.05; cluster-wise corrected).

**Conclusion:** Our results indicate the presence of DRN-prefrontal and DRN-cingulate cortex connectivity abnormalities in young medication-free depressed subjects when compared to HCs and that the severity of depressive symptoms correlates with DRN-amygdala/hippocampus connectivity. VTA connectivity with the parietal and occipital areas is related to antidepressant treatment associated with a decrease in depressive symptoms. Future studies need to be carried out in larger and different age group populations to confirm the findings of the study.

## Introduction

The brainstem monoamine nuclei (BSMN) pathways of the ventral tegmental area (VTA) which produces dopamine, dorsal raphe nucleus (DRN) which produces serotonin (5-HT) and locus coeruleus (LC) which produces norepinephrine (NE), are integral to the cognitive and emotional functioning of the brain. The three monoamines are more properly called neuromodulators, rather than neurotransmitters, as they have significant modulatory effects on cognitive, conative, and affective functions (1, 2). Depletion of monoamines has also been used to develop animal models of depression (3, 4). Antidepressants' mechanism of action has been linked to monoamine reuptake mechanisms or post-synaptic receptors' actions (5). Monoamine depletion with a tryptophan-deficient diet can lead to the precipitation of depressive symptoms and can reverse antidepressant treatment effects (1, 6). To date, however, the structural and functional aspects of monoamine pathways have not been studied *in vivo* in healthy subjects and depressed patients. In this study, we investigated the role of VTA and DRN in young adults with depression. LC was not studied as due to its small size it is much more difficult to identify it by using anatomical landmarks.

The VTA dopaminergic neurons project to cortical and limbic areas through the neocortical and mesolimbic pathways (7). The mesolimbic projections occur through the medial forebrain bundle (MFB), and project primarily to the Nucleus Accumbens (NAcc), and are thought to be the major pathways involved in the brain reward circuit (8). Deep brain stimulation (DBS) of the VTA may be the end mechanism via which antidepressant effects of DBS are hypothesized to happen (9–11). In neurodegenerative illness, Lewy bodies of the VTA have been reported to be associated with depression (12). Therefore, it has been hypothesized that structural or functional abnormalities of the VTA may occur in depression.

The Raphe Nuclei (RN) 5-HT neurons also extensively project to the cortical and limbic areas of the brain. The largest nucleus is the dorsal raphe nucleus (DRN). The DRN is located in the midbrain slightly ventral to the periaqueductal gray matter, in the midline. The ventral DRN pathways project to the limbic areas of the brain such as the amygdala and anterior cingulate cortex. In depressed patients, a significant reduction of DRN has been reported in one study (13), although contradictory findings were found in a different study (14). In post-mortem studies, 5HT1A receptor and tryptophan hydroxylase abnormalities in DRN in depressed suicidal subjects have been reported (15, 16). Brain imaging studies conducted with single photon emission computerized imaging (SPECT) and positron emission tomography (PET) have also shown presynaptic 5-HT transporter uptake abnormality in the midbrain (17, 18). In addition, one study has reported that an antidepressant response can be predicted using the PET signal of midbrain 5-HTT uptake (19–21). In another study, it was reported that baseline higher 5HT1A binding in RN but not in cortical and subcortical regions predicted antidepressant treatment response (22). SSRI treatment has also been reported to decrease 5HT1A binding (23).

Besides neurochemistry, the functional aspects of BSMNs have also been examined. The resting state functional connectivity of the DRN has been found to be altered in depression (24). In a recent study, we examined spectral dynamics of resting state fluctuations in DRN and VTA in young depressed individuals and reported spectral slowing in the VTA and DRN though only the DRN spectral slowing correlated with severity of depression (25).

In further extending our work, in this study, for the first time, we investigated DRN and VTA functional connectivity using resting state low frequency BOLD fluctuations fMRI in a large well-characterized population of medication-free young adults (ages 18–30) (which decreases the likelihood of age-related confounds) with depression at baseline. The subjects were subsequently treated with antidepressants over 3 months to ascertain treatment response. DRN and VTA were mapped, as described in our previous report, with detailed anatomical criteria using standard brain atlases and with the help of an experienced neuroradiologist (SEJ) (25).

We investigated the hypotheses of whether VTA and DRN whole-brain connectivity is decreased in young medication-free depressed subjects, and whether these abnormalities correlate with baseline depression severity. In addition, we hypothesized that baseline DRN and VTA connectivity would be related to changes in depression and anxiety scores after 3 months of antidepressant treatment.

## Methods

### Participants

Subjects (15–30 years) were recruited from the outpatient clinic at Indiana University School of Medicine and at the Cleveland Clinic, Center for Behavioral Health and by advertisement as part of a study on depression in young adults. A total of 106 depressed and 51 healthy controls were included in the study. Out of the 106 MDD subjects, 28 subjects were excluded from the analysis for the following reasons: 6 subjects did not do the scan, 6 subjects had excessive motion, 6 subjects slept during the scan, 9 subjects had poor image quality, 1 subject's resting state scan could not be acquired. Out of the 51 healthy controls (HC) 10 subjects were excluded from analysis for the following reasons: 2 subjects did not do the scan, 1 subject had excessive motion, 2 subjects' family history was revealed subsequent to screening, 3 subjects had poor image quality and processing issues, 1 subject slept during the scan, and 1 subject's scan could not be acquired. Therefore, data from 119 subjects−78 MDD and 41 HC, was included in the analysis.

All subjects were included in the study after signing an informed consent form approved by the Investigational Review Board (IRB) at Indiana University School of Medicine and at the Cleveland Clinic Foundation. Both patients and HC were paid $25 for screening and $75 for MRI scan. All subjects underwent a detailed structured diagnostic interview—Mini Neuropsychiatric Interview (MINI) that generated a DSM-IV diagnosis (26). The inclusion criteria for MDD were: (1) aged between 15 and 30 years and able to give voluntary informed consent; (2) satisfies the DSM-IV-TR criteria for MDD using a structured interview; (3) never met criteria for mania or hypomania; (4) 17-item Hamilton Depression Rating Scale score (HAM-D) (27) > 18 and < 25; (5) Young Mania Rating Scale (YMRS) (28) score < 10; (6) satisfies safety criteria to undergo an MRI scan; and (7) able to be managed as outpatients during the study, ascertained by the following—(i) Clinical Global Severity Scale < 5, i.e., moderately ill and (ii) no significant suicidal or homicidal ideation or not grossly disabled.

The exclusion criteria for all patients were: (1) satisfies the DSM-IV criteria for schizophrenia, schizoaffective disorder, or an anxiety disorder as a primary diagnosis; (2) used psychotropics in the past 2 weeks or used fluoxetine in the past 5 weeks; (3) acutely suicidal or homicidal or requiring inpatient treatment; (4) satisfies the DSM-IV criteria for substance dependence within the past year, except caffeine or nicotine; (5) positive urine toxicology screening at baseline; (6) consumed alcohol in the past 1 week or had a serious medical or neurological illness; (7) current pregnancy or breastfeeding; and (8) metallic implants or other contraindications to MRI.

The inclusion criteria for healthy subjects were: (1) aged between 15 and 30 years and able to give voluntary informed consent; (2) no history of psychiatric illness or substance abuse or dependence; (3) no significant family history of psychiatric or neurological illness; (4) not currently taking any prescription or centrally acting medications; (5) no consumption of alcohol in the past 1 week; and no serious medical or neurological illness. The exclusion criteria for healthy subjects were: (1) pregnant or breastfeeding and (2) metallic implants or other contraindications to MRI.

#### Antidepressant Treatment

After baseline assessments, all depressed subjects who wanted to be started on an antidepressant were immediately started on open-label real world treatment with an antidepressant. The default starting medication used was fluoxetine. If, for some reason, fluoxetine was contraindicated or the patient did not want to take that medication, another antidepressant was used. The antidepressant dosage was increased, or a combination of antidepressants was used, depending on response and tolerance. The goal of clinical treatment was to treat the depression adequately to achieve a euthymic or near euthymic state. For data analysis, the dose of all antidepressants used was converted to a fluoxetine-equivalent dose (29). After the start of treatment, subjects were initially followed-up on a twice-weekly basis within the first month and then on a monthly basis. Treatment response data for up to 3 months of treatment was included as that is sufficient time for an initial antidepressant treatment response. Patients were rated on the 17-item HAM-D and Hamilton Anxiety Scale (HAM-A) (30) at baseline and all follow-up time points.

### Functional MRI Acquisition

Methods used in this study are similar to that reported in our previous report of BSMN regional spectral analysis (25). Imaging data consisted of T1- and T2-weighted structural scans and resting state fMRI (RS-fMRI), as well as a brief fieldmap scan. The imaging data was acquired at the Indiana University, School of Medicine at Indianapolis using the 3T scanner from Siemens Trio MR scanner (8 MDD and 4 HC) and Cleveland Clinic Main Campus imaging center using a Siemens 3T Trio MR Scanner (Siemens AG, Berlin, Germany) with a 32 receive channel head coil and electronically transferred to the Cleveland Clinic imaging archive system. During the data collection process in Cleveland Clinic, the MR scanner underwent an upgrade from Trio to Prisma.

Scan parameters at Indiana University were as follows—Anatomic scans: high-resolution 3DMPRAGE with echo time = 2.91 ms, repetition time = 2,300 ms, inversion time = 900 ms, flip angle = 9°C, field of view = 240 × 256 mm. RS-fMRI scans: 5:44 min scan with eyes open looking at a fixation cross. 39 slices, 2.5 × 2.5 × 3.5 mm voxels, TR/TE = 2,250/29 ms. One hundred and forty five volumes were acquired.

Scan parameters at Cleveland Clinic were as follows—Anatomic scans: high-resolution 3DMPRAGE with echo time = 2.98 ms, repetition time = 2,300 ms, inversion time = 900 ms, flip angle = 9°C, field of view = 240 × 256 mm. RS-fMRI scans: 6:16 min scan with eyes open looking at a fixation cross. 39 slices, 2.5 × 2.5 × 3.5 mm voxels, TR/TE = 2,800/29 ms. One hundred and thirty two volumes were acquired. Participants at Cleveland Clinic were fitted for a bite bar to restrict head motion during scanning.

Correction for scanner type was included in the second-level analysis. All EPI data were corrected for spatial distortion using a fieldmap-based shiftmap before further analysis. This step allows for the matching of anatomy to warped regions of the brain, including the amygdala and OFC. For resting state functional connectivity, the first four volumes were discarded to ensure steady-state RF saturation. Functional connectivity image volumes were acquired while subjects were in the resting state with their eyes open, looking at a fixation cross, and instructed to think of nothing in particular. After the resting state scan was completed, the subjects were asked whether they stayed awake and complied with instructions and only those who reported complying with the instructions were included in the analysis.

### Data Analyses

#### Image Analysis

##### Pre-processing including motion correction

The images were corrected for physiologic noise (31–33) using signals obtained with PESTICA (Physiologic Estimation by Temporal ICA) (32). Special attention was paid to motion correction because both linear and non-linear motion artifacts have been shown to affect functional results (34, 35). Motion correction was performed using SLice-Oriented MOtion Correction (SLOMOCO) (36). SLOMOCO first performs an in-plane slicewise motion registration followed by an out-of-plane motion parameter estimation and regularization. The regularized out-of-plane and residual in-plane motion parameters are used in a slice-specific second-order motion model that accounts for the effect of adjacent slice motion into or out of the slice of interest as well as the present slice. Finally, the software regresses the physiologic noise model in parallel with the slice-wise second-order motion model, and this regression correction comprises the last stage of SLOMOCO to produce data that has been corrected for physiologic noise and motion.

After motion correction, images were corrected for non-neural sources of variance using a regression-based correction with time series obtained from eroded white matter and ventricular mask (37). The corrected images were normalized to Montreal Neurological Institute (MNI) space, resampled to 2 mm isotropic voxels and finally, bandpass filtered to retain low-frequency fluctuations (0.008-−0.08 Hz; the Nyquist frequency for the sampling bandwidth) using 3dBandpass, from AFNI (Analysis of Functional Neuroimages)(38). For every scan, the number of motion-corrupted volumes was identified using the Jiang average voxel displacement measurement (39) computed from the slice-wise motion parameters from SLOMOCO. A corrupted volume was defined as a volume where at least one slice within that volume experienced > 1 mm of out-of-plane motion. Any subject's scan with 13 or more corrupted volumes (i.e., more than 10% of volumes) (36, 39) was not used in the analyses.

#### Derivation of ROIs for DRN and VTA

This was done as described in our previous report (25). However, for the sake of completion, the method is described again in this report. Due to the limited contrast resolution of small brainstem nuclei in MRI images and a lack of a standard corresponding MRI atlas, DRN, and VTA regions of interest (ROIs) were identified using an operationalized anatomical criteria procedure developed by an experienced neuroradiologist (SJ) using two well-established brainstem atlases (40, 41).

The procedure has three steps: (1). Define a brainstem axis that will form the perpendicular to axial planes; (2). Define the superior and inferior extents of the DRN and VTA; (3). Manually drawing the extents of the DRN and VTA in the axial plane. The brainstem axis and axial planes are defined as perpendicular to the mid-sagittal plane and parallel to the line between the central mammillary bodies and the intercollicular fossa. The Paxinos atlas is used to determine the superior and inferior extents because of its uniform axial spacing and well-marked measured longitudinal reference of the axial image with respect to the obex (Paxinos, figures 50–59) (40). For the DRN, we defined the inferior extent as the plane containing the inferior edge of the inferior colliculus (IC) and the superior extent as the plane halfway between the central superior colliculus (SC) and the intercollicular fossa—a superior-inferior distance of typically 7 mm. For the VTA, we defined the inferior extent as the plane containing the center of the IC and the most superior extent as the plane containing the center of the superior colliculus (SC)—a superior-inferior distance of typically 6 mm. We used the Duverney atlas to draw the DRN and VTA ROIs in the axial plane, as the Paxinos atlas has more detail than can be seen in the MRIs (Duverney chapter 4, page 55; and figures 2.18–2.20) (41). In each axial slice, the midline periaqueductal gray matter (PaqGM) is marked off as a strip 1 mm anterior to the cerebral aqueduct. Extending anteriorly from the PaqGM toward the interpeduncular fossa (IpF), all the voxels on either side of the midline are split between the DRN and VTA. For the superior two thirds of DRN axial slices, the posterior two thirds of midline voxels between the IpF and PAq are DRN. For the inferior one third of DRN slices, the posterior half of midline voxels are DRN. Conversely, for the superior two thirds of VTA axial slices, the anterior one third of voxels between the IpF and PAq is VTA. For the inferior one third, the anterior half of midline voxel is VTA. Regarding the lateral extent of the DRN, for the inferior two thirds of DRN axial slices, a second line of DRN voxels are laterally added to the para-midline voxels from the most posterior DRN voxel to one third (anteriorly) up the midline strip of DRN voxels. Regarding the lateral extent of the VTA, in all axial slices, the VTA extends diagonally along the IpF border. The most superior slice extends 1 voxel, the next extends 2 voxels, and the remaining slices extend 3 voxels. A second row of VTA voxels adjoins lateral and parallel to the first diagonal along the IpF border for the inferior two thirds of axial VTA slices.

#### Region of Interest Templates and Time Course Extraction

As the method for identifying DRN and VTA is very time consuming (5–6 h per subject), these ROIs were individually mapped for each of the 17 healthy subjects. Next, the overlapping area, from these 17 healthy subjects, was used as the template for DRN and VTA, respectively and applied to the rest of the samples. First, these templates were normalized to MNI space using Statistical Parametric Mapping (SPM) version 12 software. Next, the normalized ROIs were used to extract resting state time series from all subjects.

#### First-Level Analysis for the Generation of Connectivity Maps

**Figure 1:**
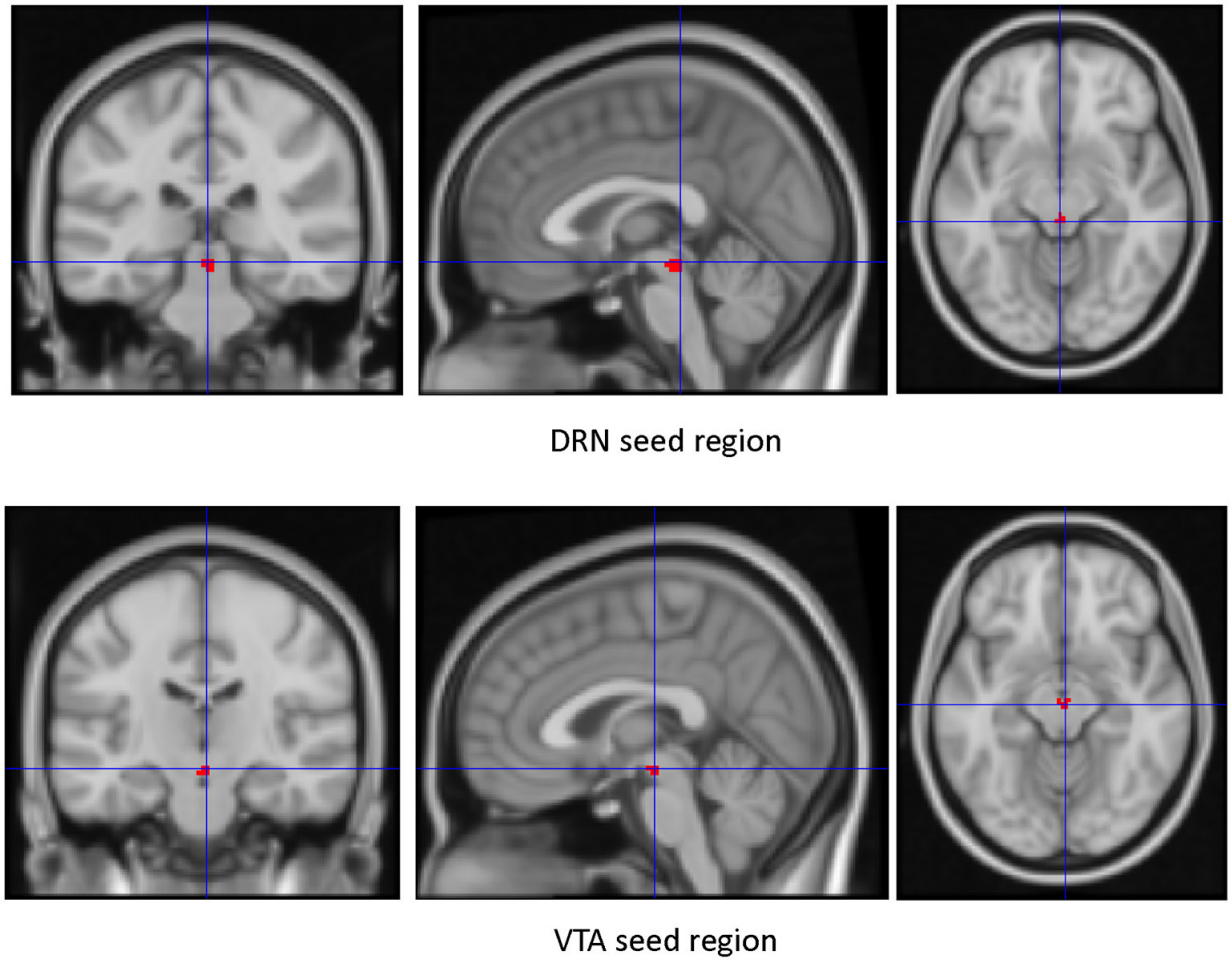
Seed ROIs used for dorsal raphe nucleus (DRN) and Ventral Tegmental Area (VTA).

Seed Regions of Interest (ROIs) (Figure 1)—The VTA and DRN ROIs generated were used as regions of interest to create whole-brain connectivity maps. The mean time series of each of the ROIs for each subject was extracted from the filtered preprocessed image. Next, by correlating the ROI time series with the rest of the brain, whole-brain resting state functional connectivity map for each subject was generated. The first level connectivity map for each subject was then Z-transformed (Fisher's Z) and smoothed with 8 mm kernel using SPM12. The resultant smoothed image was used in the second-level group analysis.

#### Second-Level Group Analysis

A second-level analysis was conducted in SPM12 using factorial analysis. The main effect for group was examined while controlling for effects of age and gender. In addition, we used scanners (two scanners and the upgrade of the second scanner) as a factor and regressed out the effect of the scanner. This is a statistically rigorous way to control for differences in scanner effects. The main effect for the group was examined at a voxel-wise threshold of *p* = 0.01 (uncorrected) (42) using the whole brain mask. Cluster level significant results at *p* = 0.05 (corrected) were then calculated using the 3Dclustsim tool in AFNI using the autocorrelation function (ACF) method (42). Next, the main effects for ROIs were used to extract connectivity values and used in a statistical software (SPSS ver. 21) to depict the differences between groups as box plots.

#### Treatment Response and Correlation of Change in Depression Scores to Clinical Improvement

Percentage changes in scores of 17-item HAM-D and HAM-A was correlated with baseline DRN and VTA connectivity maps. Additionally, the percentage change in scores was correlated with clusters significantly different at baseline between the MDD and the HC groups.

## Results

Data from 41 healthy subjects and 78 depressed subjects was included in the analyses. The demographic and clinical characteristics of the study population are presented in Table 1. The ratio of subjects in each group for each of the two scanners was similar. For the first scanner, 8 MDD and 4 HC subjects were studied (ratio: 2) and for the second scanner, 70 MDD and 37 HC subjects were studied (ratio 1.89). The mean age of healthy subjects was 24 ± 3 years whereas the mean age of depressed subjects was 24 ± 4 years.

**Table 1:** Illness characteristics and demographics.

**Illness characteristics**	**MDD** **(*N* = 78)**	**Healthy** **(*N* = 41)**	***p*-value**
Age (years) [mean (SD)]	24(4)	24(3)	ns
Female [*n* (%)]	54 (69%)	24 (59%)	ns
Caucasian [*n* (%)]	67 (86%)	31 (76%)	0.013
African American [*n* (%)]	10 (13%)	4 (10%)	
Asian [*n* (%)]	1 (1%)	6 (14%)	
Education (years) [mean(SD)]	15(2)	17(2)	< 0.001
HAM-D 17 item [mean (SD)]	18(3)	0(1)	
YMRS [mean (SD)]	1(2)	0 (0)	
HAM-A [mean(SD)]	15(6)	1(1)	
Age at first episode (years) [mean (SD)]	14(4)		
Medication free period (weeks) [mean (SD)]^*^	85 (95)		
Number of depressive episodes [mean (SD)]^**^	27(31)		
History of psychosis [*n* (%)]	5(6)		
Scanner 1 (*n*)	8	4	
Scanner 2 (*n*)	26	7	
Scanner 2 upgrade (*n*)	44	30	
Slicewise mean motion [mean(SD)]	0.260 (0.06)	0.245 (0.059)	ns
Volumetric mean motion [mean(SD)]	0.344 (0.11)	0.346 (0.13)	ns

* 31 subjects were treatment naive, the mean and SD values are for the remaining 47 subjects.

** *4 subjects have missing information*.

Out of the 78 subjects, 47 subjects who completed 3 months of antidepressant treatment were included. The mean starting dose of fluoxetine-equivalent medication in these 47 depressed subjects was 13 ± 5 mg and the mean end dose was 35 ± 16 mg. The total average dose of fluoxetine-equivalent dose at the end of 3 months was 28 ± 11 mg. Out of 47 subjects, 29 were only given fluoxetine and the rest were given or changed to another serotonin reuptake inhibitor. For just one person, bupropion was added to augment the effects of fluoxetine.

### Baseline Differences Between Depressed and Healthy Subjects

**Figure 2:**
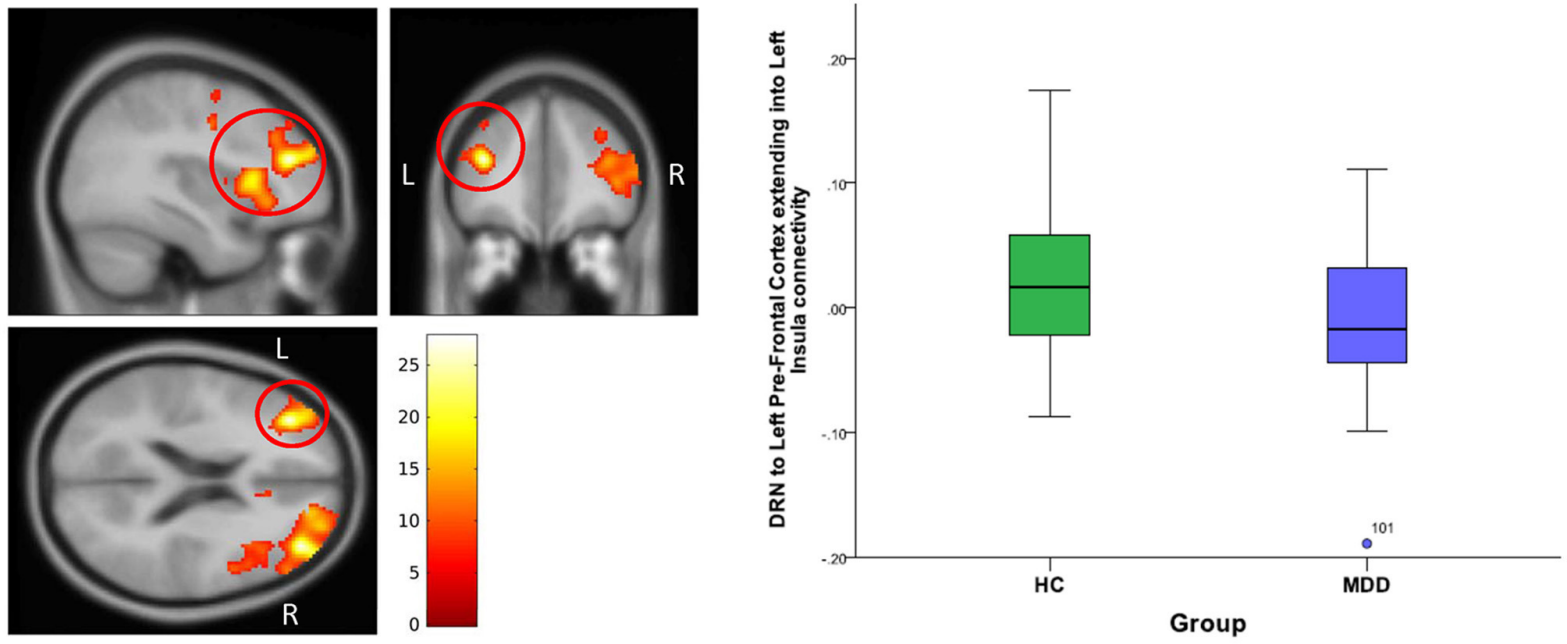
DRN to left pre-frontal cortex extending into Left Insula connectivity differences between HC and MDD. The cluster-wise significance threshold was set at *p* < 0.01(uncorrected), *k* = 1,143 voxels corresponding to *p* < 0.05 (corrected).

**Figure 3:**
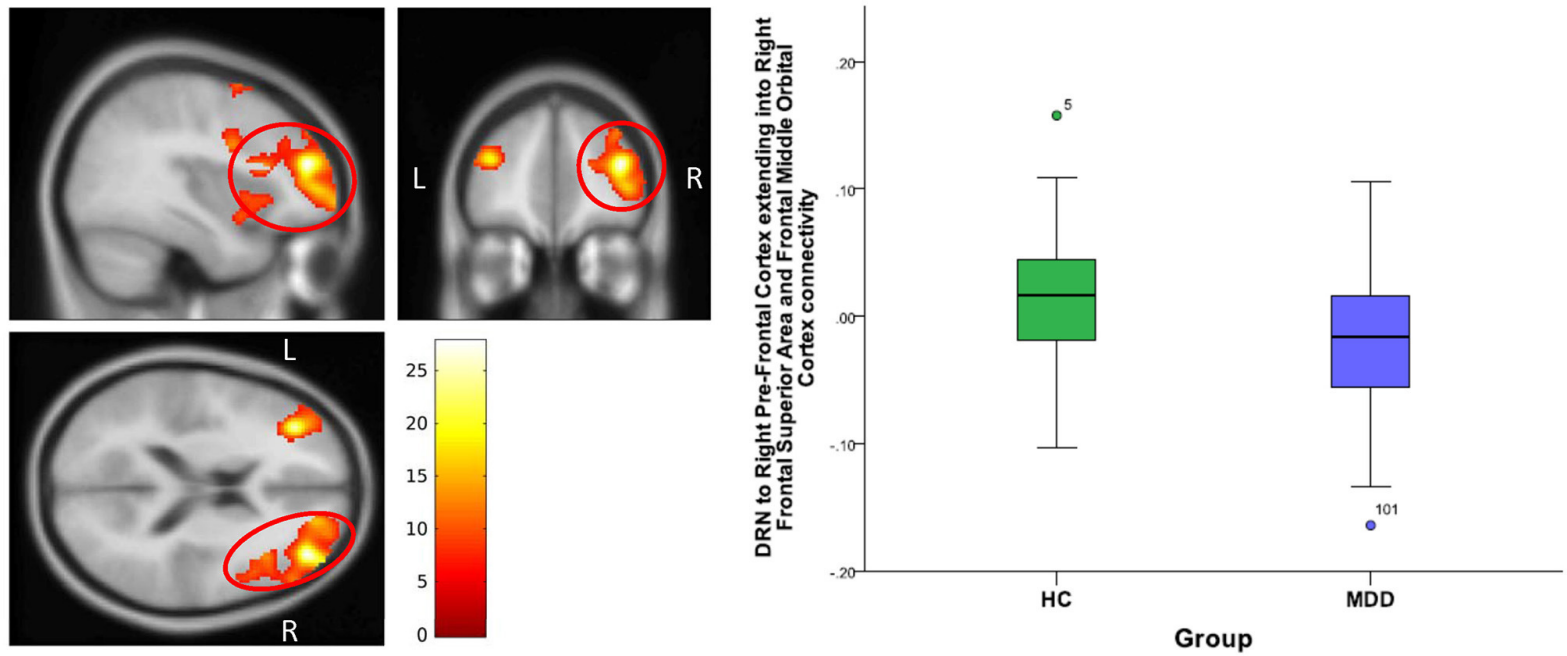
DRN to right pre-frontal cortex extending into right frontal superior cortex and frontal middle orbital cortex connectivity differences between HC and MDD. The cluster-wise significance threshold was set at *p* < 0.01 (uncorrected), *k* = 1,143 voxels corresponding to *p* < 0.05 (corrected)|.

**Figure 4:**
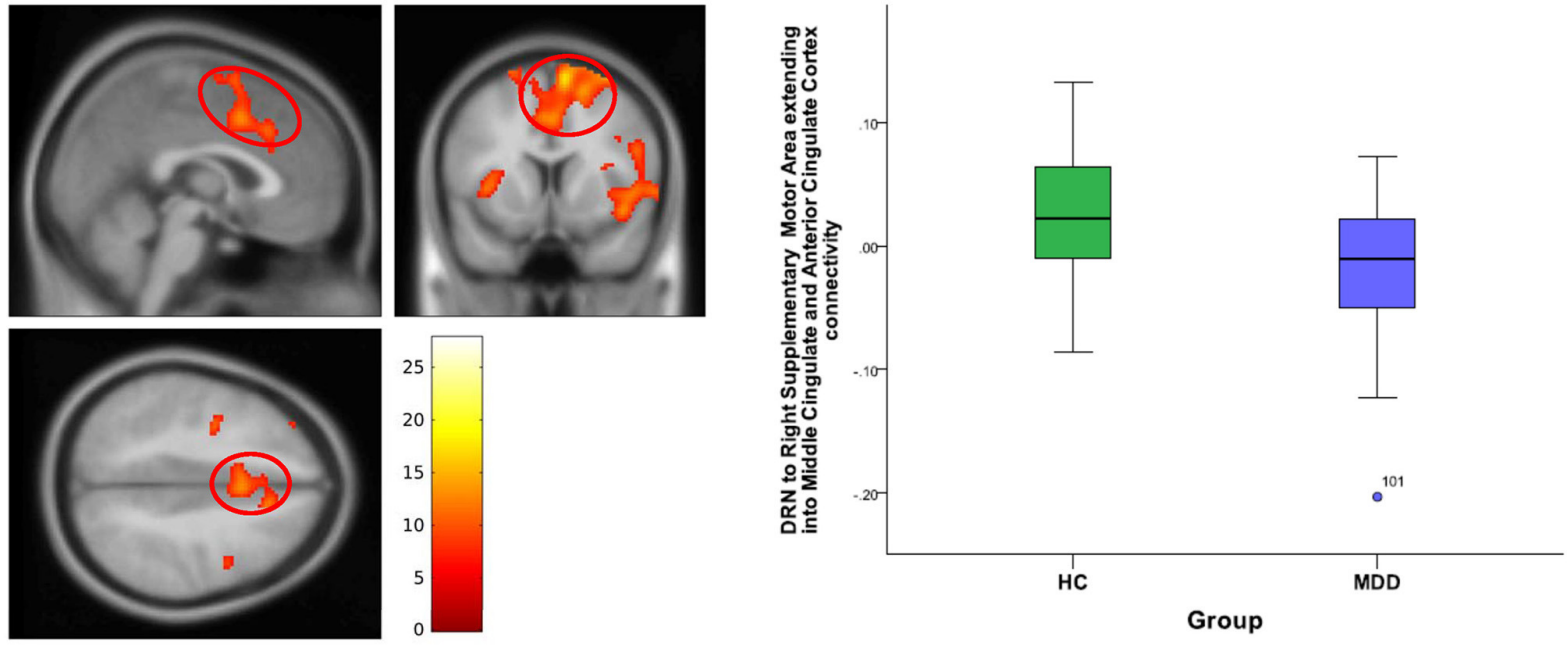
DRN to right supplementary motor area and anterior cingulate cortex connectivity differences between HC and MDD. The cluster-wise significance threshold was set at *p* < 0.01 (uncorrected), *k* = 1,143 voxels corresponding to *p* < 0.05 (corrected).

**For DRN-whole brain connectivity**, the MDD group showed decreased functional connectivity compared to the HC group in terms of DRN connectivity with large bilateral clusters in the prefrontal cortex extending caudally into the orbitofrontal cortex (peak MNI coordinates: (*x* = 38; *y* = 46; *z* = 18), *F* = 27.73, df = (1,111), *Z* = 4.83; and at (−36, 40, 20), *F* = 26.33, df = (1,111), *Z* = 4.71) (Table 2, Figures 2–4). Another area of decreased functional connectivity was located in the right supplementary motor area extending medially into the mid cingulate and anterior cingulate (peak MNI coordinates(14, 2, 68), *F* = 26.32, df = (1,111), *Z* = 4.71).

**Table 2:** Main effect of group.

**ROI**	**Target area**	**Cluster size**	**Peak Z**	**Peak *p*(uncorrected)**	**x,y,z (MNI)**
DRN	Right pre-frontal cortex extending into right frontal superior area and left frontal middle orbital cortex	3,773	4.83	0.0001	38,46,18
DRN	Left pre-frontal cortex extending into left insula	1,688	4.71	0.0001	−36,40,20
DRN	Right supplementary motor area extending into middle cingulate and anterior cingulate cortex	3,287	4.71	0.0001	14,2,68

*The cluster-wise significance threshold was set at p < 0.01 (uncorrected) and cluster size of 1,143 voxels corresponding to p < 0.05 (corrected)*.

**Figure 5:**
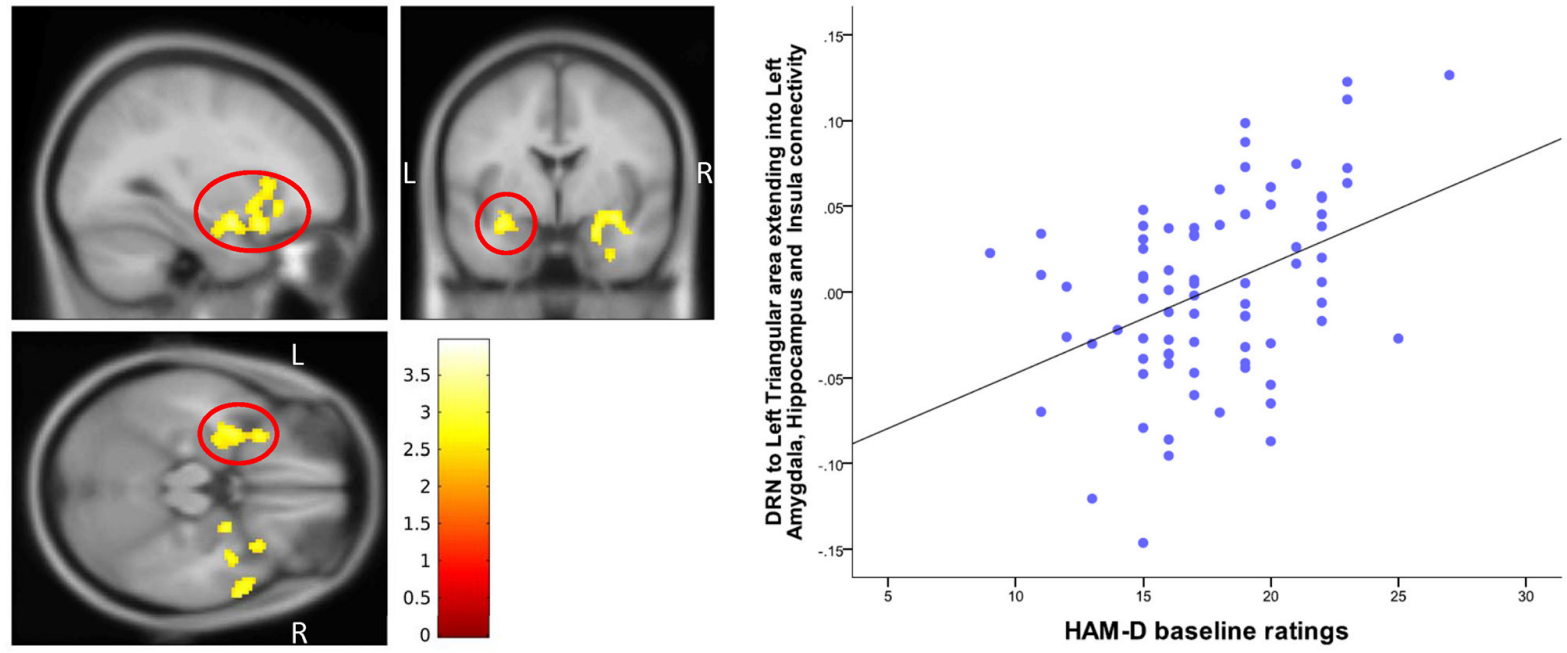
Correlation of baseline HAM-D scores with DRN-Temporal lobe connectivity. The cluster-wise significance threshold was set at *p* < 0.01 (uncorrected), *k* = 1,205 voxels corresponding to *p* < 0.05 (corrected).

A positive correlation of 17-item HAM-D scores was found for DRN connectivity with left inferior bilateral temporal lobe clusters, which involved the amygdala and the hippocampus and extended into the insula (peak MNI coordinates (−48, 24, 6), *T* = 3.95, df = (1,74), *Z* = 3.75 and peak MNI coordinates (50, 20, −30), *T* = 3.53, df = (1,74), *Z* = 3.38) (Table 3, Figure 5).

**Table 3:** Correlation between DRN connectivity and HAM-D scores.

**ROI**	**Target area**	**Correlation**	**Cluster size**	**Peak Z**	**Peak *p*(uncorrected)**	**x,y,z (MNI)**
DRN	Left Inferior triangular area extending into left amygdala, hippocampus, left insula and inferior OFC	HAM-D positive	1,455	3.75	0.0001	−48,24,6
DRN	Right temporal middle pole extending into right amygdala and right insula	HAM-D positive	1,404	3.38	0.0001	50,20,−30

*The cluster-wise significance threshold was set at p < 0.01 (uncorrected) and cluster size of 1,205 voxels corresponding to p < 0.05 (corrected)*.

On further examination, we examined the differences between the two groups within the hippocampus/amygdala/insula cluster (Table 3) found to correlate with baseline HAM-D scores. DRN connectivity showed a significant main effect of group (*p* = 0.05 cluster-wise corrected) within the hippocampus/amygdala cluster (peak MNI coordinates (−34, 18, 4), *F* = 18.3, *df* = (1,111), Z = 3.94).

In the same vein, we went back and examined the correlation of baseline HAM-D ratings with DRN connectivity to each of the three ROI clusters which showed differences between the depressed and healthy subject groups (Table 2). Baseline 17-item HAM-D scores of the depressed subjects showed a significant (*p* = 0.05 cluster-wise corrected) correlation within an area of the DRN-left frontal cortex/insula connectivity (peak MNI coordinates (−36, 24, 4), *T* = 3.45, *df* = (1, 74), *Z* = 3.31).

**For VTA-whole brain connectivity**, no differences between the two groups were found and no correlation was found with the depression and anxiety scale scores.

### Treatment Effect and Response

**Figure 6:**
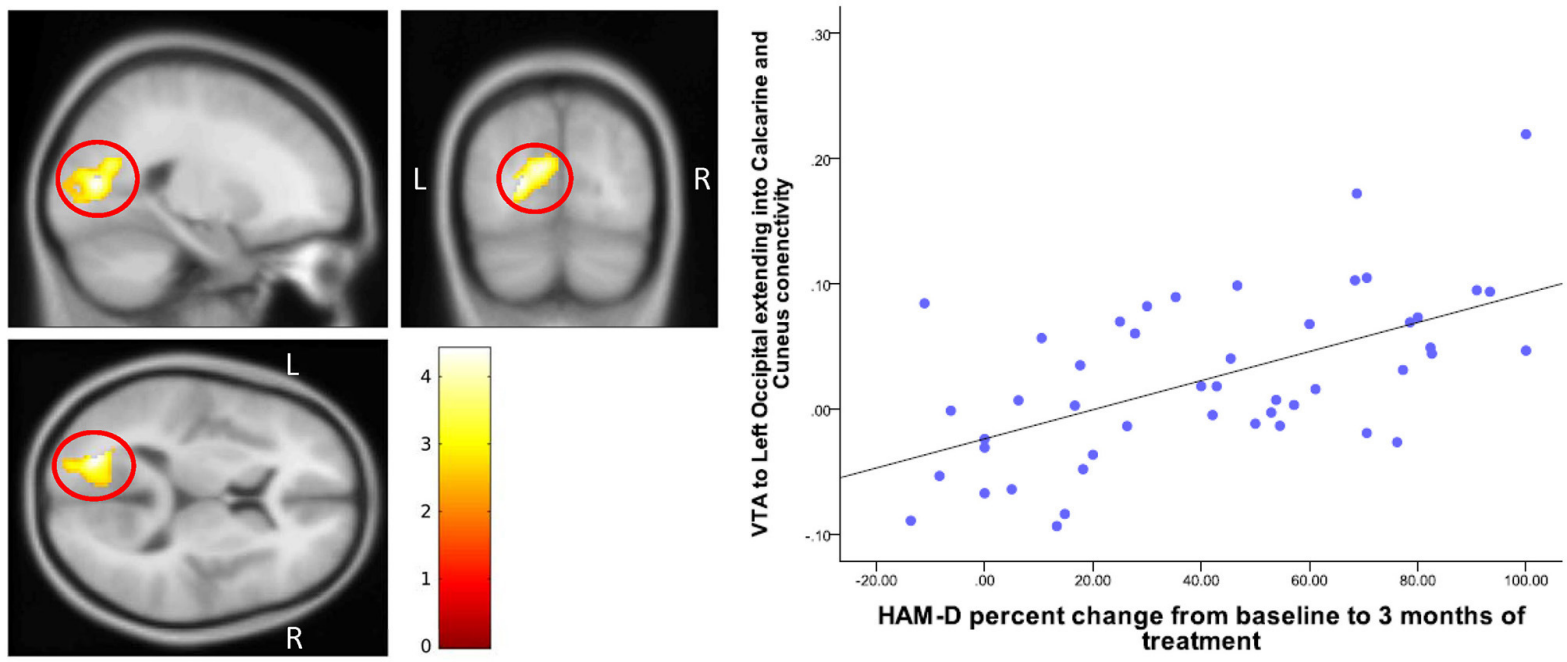
Correlation of change in HAM-D scores with VTA-Cuneus connectivity. The cluster-wise significance threshold was set at *p* < 0.01 (uncorrected), *k* = 1,116 voxels corresponding to *p* < 0.05 (corrected).

Baseline DRN connectivity was not significantly correlated with either change in 17-item HAM-D scores or HAM-A scores. Baseline differences between MDD and HC in DRN- prefrontal and DRN-cingulate cortex connectivity also did not correlate with a change in depression scores. Baseline VTA connectivity with the left cuneus-occipital area, however, correlated with a greater percentage decrease in HAM-D scores from baseline (peak at MNI coordinates (−22, −76, 10), *T* = 4.40, *df* = (1, 43), *Z* = 3.98) (Table 4 and Figure 6).

**Table 4:** Correlation between VTA connectivity and antidepressant treatment associated percent change in 17-item-HamD scores.

**ROI**	**Target area**	**Correlation**	**Cluster size**	**Peak Z**	**Peak *p*(uncorrected)**	**x,y,z (MNI)**
VTA	Left cuneus, occipital, calcarine	HAM-D positive-treatment effect	1,146	3.98	0.0001	−22,−76,10

*The cluster-wise significance threshold was set at p < 0.01 (uncorrected) and cluster size of 1,116 voxels corresponding to p < 0.05 (corrected)*.

## Discussion

The findings of this study indicate that MDD subjects show a decreased connectivity of the DRN with the prefrontal and cingulate cortices compared to healthy subjects, but VTA connectivity was not different. The severity of depression in MDD subjects correlated with DRN connectivity with limbic areas bilaterally encompassing the amygdala, hippocampus, and the insula. VTA connectivity did not correlate with depression severity, although VTA-cuneus connectivity correlated with a change in depression scores after antidepressant treatment.

The findings of this study, therefore, support the role of the monoamine function in depression, with a possible decrease in serotonergic input to the prefrontal and cingulate cortex in depression. Blood flow, activation, and functional connectivity studies (43, 44) have described prefrontal and cingulate cortex abnormalities in depression. The prefrontal and cingulate cortices are responsible for higher order functions such as abstraction, memory, and emotional regulation, which are frequently altered in depression. The prefrontal and cingulate cortices receive a large amount of innervation from the dorsal raphe and a number of presynaptic and postsynaptic serotonin receptors are expressed in these areas (45). The findings of this study suggest that decreased functional connectivity between DRN and prefrontal/cingulate cortex may underlie the pathophysiology of depression and may be the basis of altered cognition and emotional regulation seen in this illness.

The finding of an association between severity of depression and increased DRN connectivity with the temporal lobe limbic structures such as the amygdala and the hippocampus is of interest. DRN input into the amygdala and other limbic structures has been implicated in the modulation of anxiety and fear response (46). However, the direction of modulation has not been fully elucidated, with some studies reporting increased anxiety related to DRN activation and others noting an inhibitory effect of serotonergic function on amygdala-activation induced fear (47). Increased DRN-temporal lobe structures connectivity in our studies suggests that increased connectivity may be related to depression symptoms.

Regarding treatment effect, no correlation was seen between DRN connectivity and either the change in HAM-D scores or HAM-A scores from baseline. The positive correlation of treatment response as measured with the decrease in HAM-D score and VTA-parietal cortex connectivity is a new finding. Other investigators have reported cuneus connectivity with limbic regions being altered in depression and an increased VTA-cuneus and VTA-occipital cortex connectivity in patients treated with SSRIs (21). The cuneus is also part of the salience network whose connectivity has been reported to be altered in depression and related to treatment effects (48–50). We did not find any significant correlation to treatment response in the regions that showed baseline group differences. One explanation is that antidepressant treatment may be acting on different regional connectivities which compensate for the underlying connectivity abnormality in depression. It is well-known in pharmacological literature that pathophysiology of the disease and the mechanism of action of treatment can be different. For example, the mechanism of action of antidepressants i.e., an increase in monoamine neurotransmission, does not necessarily translate into a monoamine deficiency in depression.

There are several strengths of this study, of which the most important is the inclusion of medication-free subjects. Moreover, as most of the subjects were young, within a narrow age range of 15-−30, the effects of confounding effects of chronic psychiatric or medical illness, as well as age, were mitigated. Another strength was that the antidepressant response was measured prospectively.

Limitations of the study include the small ROIs for DRN and VTA. We used a detailed rigorous method developed by a senior experienced neuroradiologist (SJ), using two gold-standard atlases (40, 41), to identify DRN (keeping in mind its shape) and VTA. Using this method, the volume of the bilateral DRN ROI is 128 mm^3^, corresponding to around 6 voxels in the acquired image and the volume of bilateral VTA is ~ 112 mm^3^, corresponding to 5 voxels. As these areas are small, the ROIs are bound to be small but similar small areas have been used in fMRI analysis of other parts of the brain e.g., nucleus accumbens or amygdala subnuclei. The use of small homogenous regions can be an advantage as ROIs of large areas used in fMRI studies are very heterogeneous regarding underlying anatomy and the subareas contained may have different functions. The ROIs were drawn on a subset of 17 healthy subjects, which could have led to a bias in terms of differences seen between the patient and the healthy groups. Using our method of normalizing these ROIs to standard (MNI) space and then applying them on individual scans, we checked the location of the ROIs for each subject individually in both the healthy and the depressed groups to minimize any bias that may have occurred due to ROIs being originally drawn only on a subset of healthy subjects.

Another limitation of the study is that a specific population group was studied and future studies need to be conducted in older populations. Though we controlled for age and gender, other confounds such as comorbid conditions such as anxiety disorder, subthreshold bipolar symptoms, or psychotic symptoms, were not controlled for what may need to be accounted for in future studies. The antidepressant treatment given was naturalistic in nature and open-labeled; therefore, placebo effects cannot be fully accounted for. However, placebo-controlled imaging studies are difficult to justify in patients suffering from acute depression. Furthermore, even though all subjects were not given one single antidepressant, the results of the study are applicable to the real-world clinical treatment of depressed subjects, which was the aim of the study. Most subjects were given the antidepressant fluoxetine, and another antidepressant was only given or added if there were tolerability or efficacy issues.

## Conclusion

The findings of this study identified DRN-frontal cortex connectivity abnormalities in young medication-free depressed subjects compared to HCs and found that the severity of depressive symptoms correlated with the DRN-temporal lobe structures connectivity. This study also identified VTA connectivity with the parietal areas as related to a decrease in depressive symptoms. Future studies need to be carried out in larger and different age group populations to confirm the findings of the study.

## Author Contributions

AA was involved in the conception and implementation of the study as well as data analysis, interpretation and manuscript submission. ML and SJ were involved in imaging acquisition and analysis methodology and manuscript publication. HK was involved in data and imaging analysis. PK was involved in preparation of results and manuscript.

### Conflict of Interest Statement

The authors declare that the research was conducted in the absence of any commercial or financial relationships that could be construed as a potential conflict of interest.
