# Supplementary material for: Resting State Functional Connectivity of Dorsal Raphe Nucleus and Ventral Tegmental Area in Medication-Free Young Adults With Major Depression
Source: Front Psychiatry. 2019 Jan 25;9:765. doi: 10.3389/fpsyt.2018.00765 (PMC6362407; doi:10.3389/fpsyt.2018.00765)
Supplement: Supplementary file 1 [file Data_Sheet_1.PDF]

SUPPLEMENTARY TABLES For Comparable results for subjects imaged on Scanner 2 only (MDD: 70, Healthy Controls 37).

Supplementary Table 1: Main effect of group without subjects from Scanner I (Indiana University).The results shown below were examined at voxel-wise p< 0.01 (uncorrected). For cluster-wise significance threshold for p < 0.05 (corrected) a cluster size of 1128 voxels is needed.

• ROI	Target area	cluster size	peak Z	peak p(uncorrected)	x,y,z (MNI)
DRN	Right Pre-Frontal Cortex extending into Right Frontal Superior Area and Left Frontal Middle Orbital Cortex	728	3.6	0.0001	36 64 -6
DRN	Left Pre-Frontal Cortex extending into Left Insula	396	3.69	0.0001	-38 20 12
DRN	Right Supplementary Motor Area	304	4.01	0.0001	14 2 66
DRN	Left Ventral Anterior Cingulate Cortex extending into Caudate	781	3.52	0.0001	-2 10 -8

Supplementary Table 2: Correlation between DRN connectivity and HAM-D scores without subjects from Scanner I (Indiana University). The results shown below were examined at voxel-wise p <0.01 (uncorrected). For cluster-wise significance threshold for p < 0.05 (corrected) a cluster size of 1216 voxels is needed. * significant

ROI	Target area	Correlation	cluster size	peak Z	peak p(uncorrected)	x,y,z (MNI)
DRN	Left Inferior Triangular Area extending into Left Amygdala, Hippocampus, Left Insula	HAM-D positive	1165	3.71	0.0001	-48 24 6
DRN	Right Temporal middle pole extending into Right Amygdala and Right Insula	HAM-D positive	1441*	3.39	0.0001	50 20 -28

Supplementary Table 3: Correlation between VTA connectivity and antidepressant treatment associated percent change in 17-item-HAM-D scores without subjects from Scanner I (Indiana University). The results shown below were examined at voxel-wise p <0.01 (uncorrected). For cluster-wise significance threshold for p < 0.05 (corrected) at p a cluster size of 1107 voxels is needed. . * significant

ROI	Target area	Correlation	cluster size	peak Z	peak p(uncorrected)	x,y,z (MNI)
VTA	Left Cuneus, Occipital, Calcarine	HAM-D positive-Treatment effect	1351*	4.03	0.0001	-20 -78 10
