# Supplementary figures and images for: Resting State Functional Connectivity of Dorsal Raphe Nucleus and Ventral Tegmental Area in Medication-Free Young Adults With Major Depression
Source: Front Psychiatry. 2019 Jan 25;9:765. doi: 10.3389/fpsyt.2018.00765 (PMC6362407; doi:10.3389/fpsyt.2018.00765)

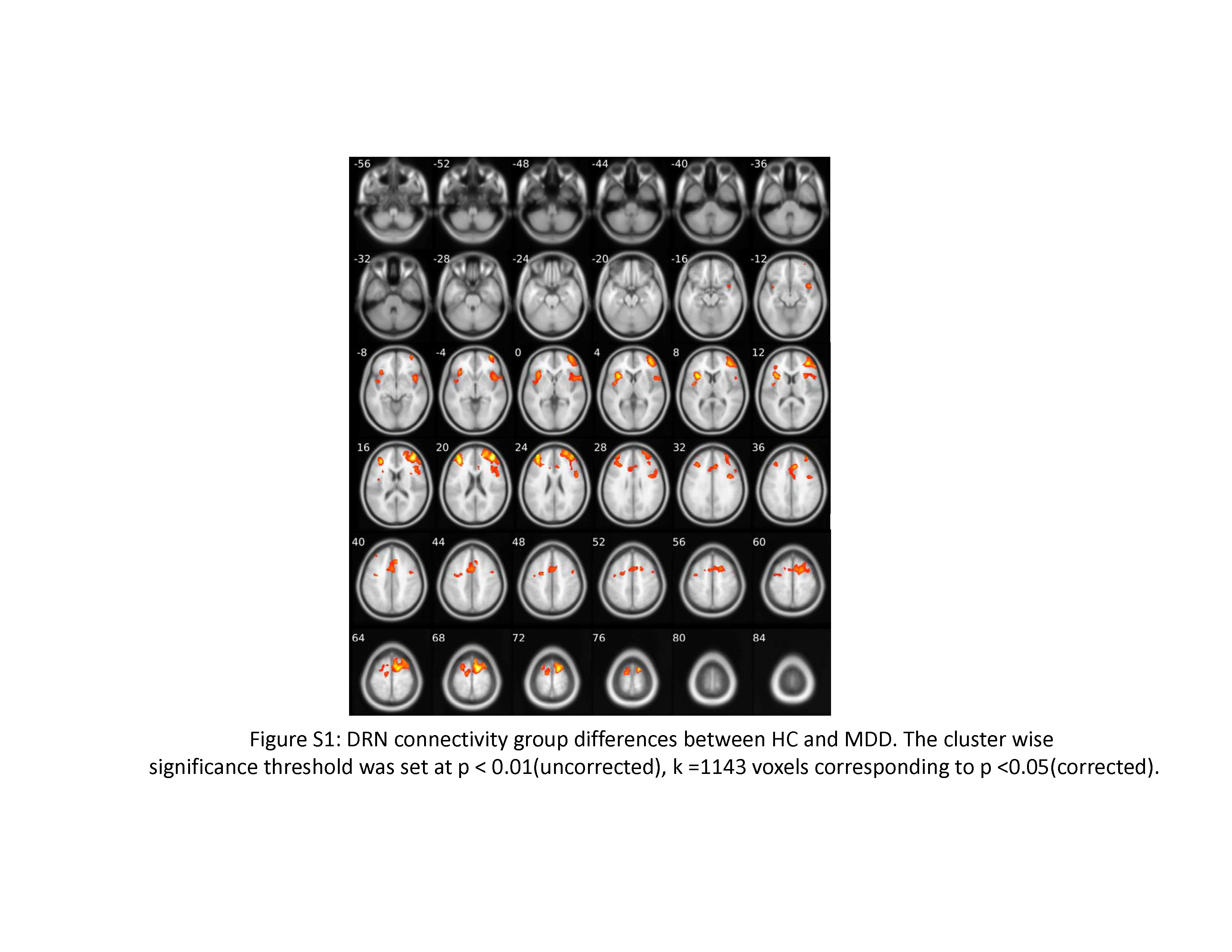

Supplement: Supplementary file 2 [file Image_1.JPEG]

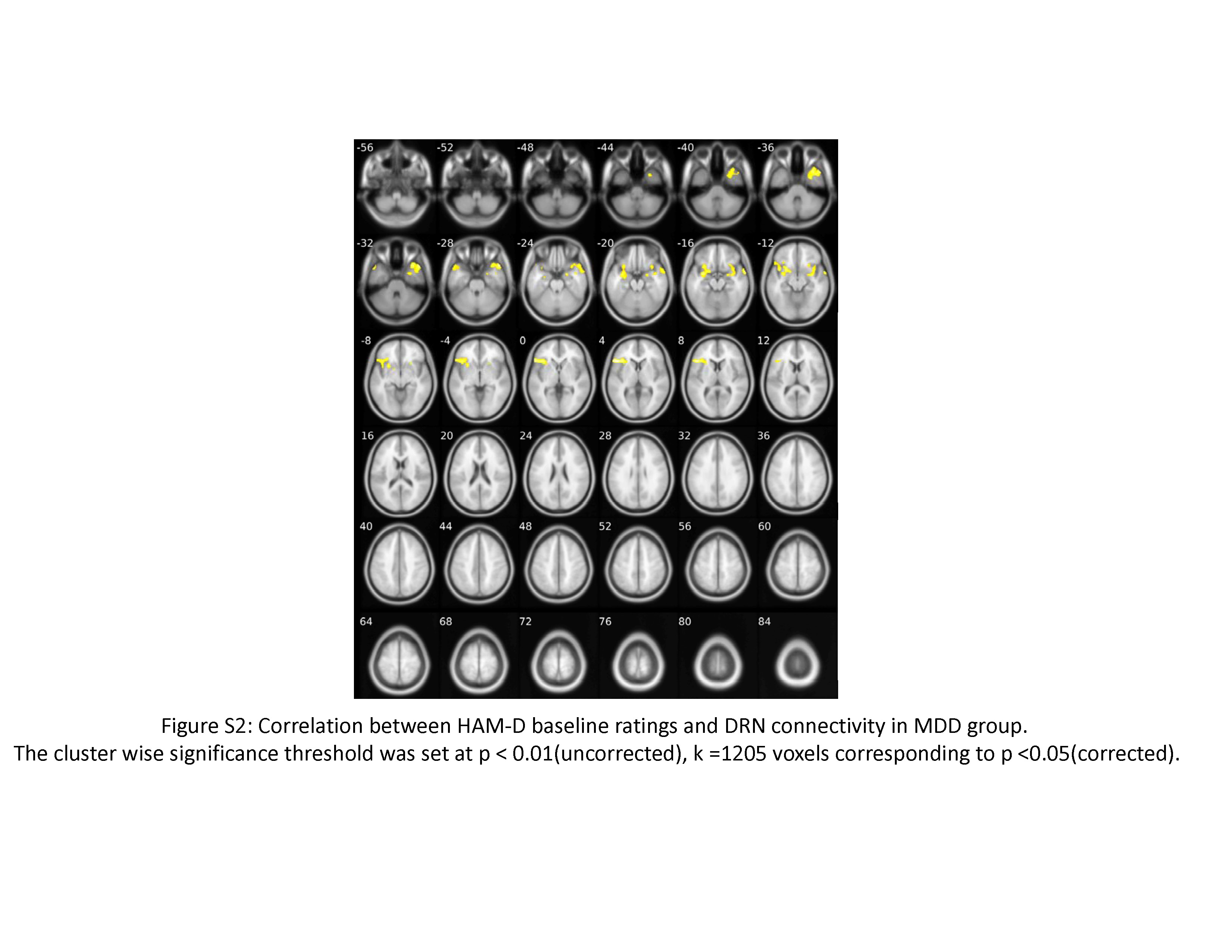

Supplement: Supplementary file 3 [file Image_2.JPEG]

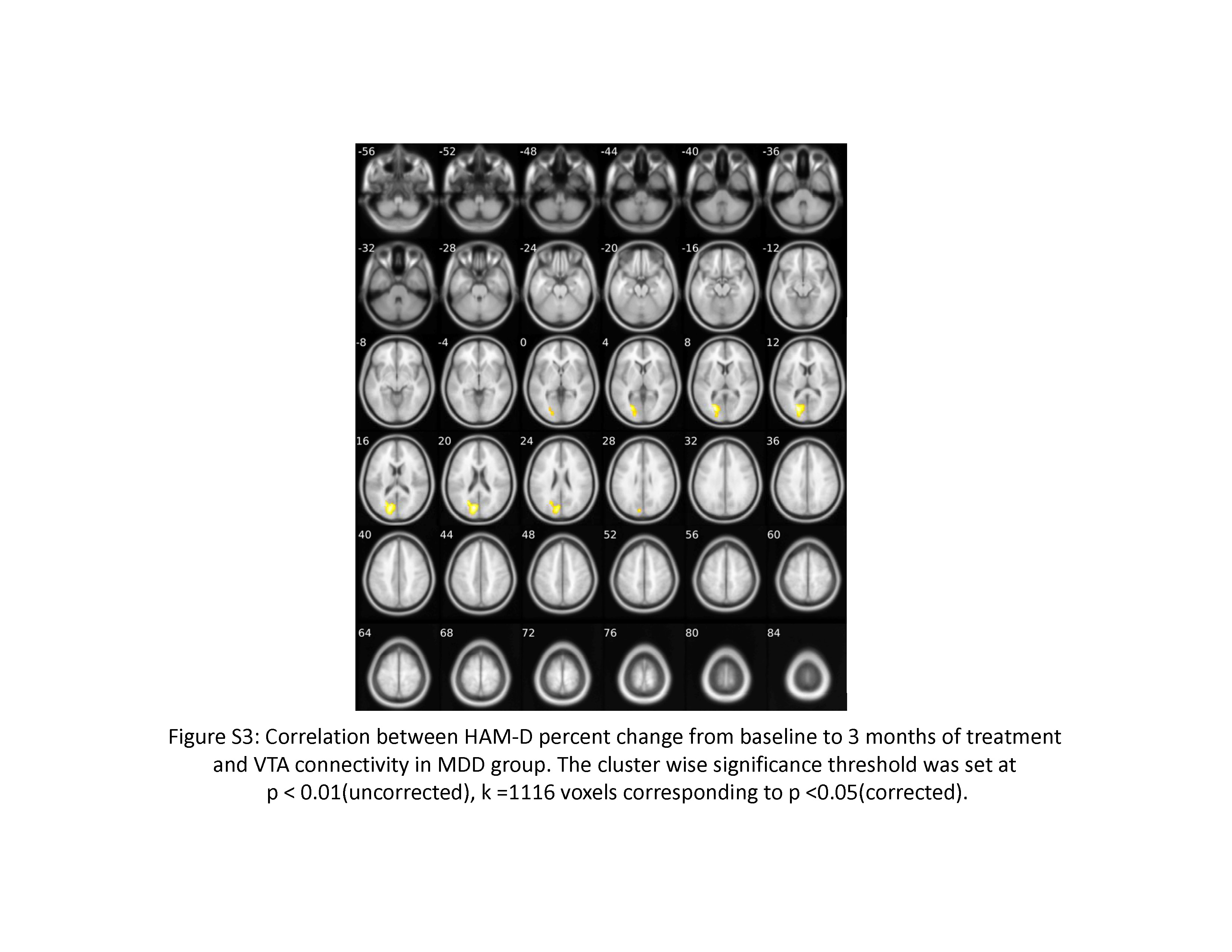

Supplement: Supplementary file 4 [file Image_3.JPEG]
